# Timing Market Entry: The Mediation Effect of Market Potential

**DOI:** 10.1177/1069031X211068072

**Published:** 2021-12-23

**Authors:** Towhidul Islam, Nigel Meade, Ashish Sood

**Affiliations:** Towhidul Islam is Professor and University Research Leadership Chair, Department of Marketing and Consumer Studies, 3653 University of Guelph, Canada (email: islam@uoguelph.ca).; Nigel Meade is Emeritus Professor, 4615 Imperial College Business School, London, UK (email: n.meade@imperial.ac.uk).; Ashish Sood is Associate Professor, 8790 University of California, Riverside, USA (email: asood@ucr.edu).

**Keywords:** market entry timings, machine learning, macroeconomic attractiveness, market potential, mobile broadband phones

## Abstract

Timing a multinational firm's entry into a new country is a pivotal decision with long-term impact on the firm's overall performance; thus, a deeper understanding of the drivers of the decision and their interrelationship can yield significant managerial benefits. The authors explore the mediating role of market potential by decomposing the total effects of the decision's main drivers—macroeconomic attractiveness, market concentration, social heterogeneity, and population density—into direct and indirect effects. These decompositions explain the countervailing effects of some drivers that simultaneously make both positive and negative impacts. The data set encompasses mobile 4G broadband penetration in 130 countries, including market entry timings for 28 international operators in 79 countries. The authors establish the nature of the mediation effect of market potential on the drivers of entry timing. Using early penetration data, they utilize growth mixture modeling to divide the countries into four latent segments. They validate this segmentation using machine learning with the four key drivers as classifiers; the process establishes macroeconomic attractiveness as the predominant classifier. The analysis offers entry-timing guidance at both pre- and postlaunch stages.

Decisions to enter foreign markets are risky and have long-term impacts on a firm's performance (Lieberman and Montgomery 2013; Zachary et al. 2014). An understanding of the drivers of market growth and their variability across countries brings significant managerial benefits for international market selection, including better allocation of resources to manufacturing, distribution, pricing, and inventory management (Islam and Meade 2018). The literature in international business (IB) and international marketing (IM) identifies the main drivers of market entry times as macroeconomic attractiveness (e.g., Mitra and Golder 2002), market concentration (e.g., Bayus, Kang, and Agarwal 2007), social heterogeneity (e.g., Berry, Guillén, and Zhou 2010), and population density (e.g., Van Everdingen, Fok, and Stremersch 2009). The market potential of each country is also recognized as one of the most important drivers of market selection (e.g., Zachary et al. 2014). In the IM literature, Gaston-Breton and Martin (2011) note that a country's macroeconomic indicators are positively correlated with market potential across different product categories and industries. It follows that market potential may be an endogenous intermediate variable between the main drivers of market entry and market entry timing, rather than an exogenous variable as typically assumed in the literature. This possible mediating effect of market potential in the relationships between the macroeconomic, market, social, and population-based drivers and market entry timing has yet to be investigated.

By addressing this issue, we respond to the research agendas of both the IB and IM literature and extend theory on international market entry decision making. To understand the process linking these drivers, Stahl and Tung (2015) suggest capturing their countervailing effects by integrating the positive and negative effects of social heterogeneity and identifying the mechanisms, such as mediation, through which social heterogeneity and cultural diversity impact market entries. In the IM literature, Islam and Meade (2018) note that in some cases, most of a driver's effect on the outcome is delivered via an intermediate variable. Further, Zachary et al. (2014) note that testing the effect of a driver is invalid without considering the relationship with other drivers. Björkman, Stahl, and Vaara (2007) show that the impact of cultural differences on foreign market entry is mediated by several intermediate variables.

The payoff of this analysis is that mediation can capture the underlying mechanism of how the main drivers identified by prior literature affect market entry decisions. Identification of this mechanism (positive, negative, or curvilinear) improves our theoretical understanding of the role of the main drivers in influencing outcomes of interest such as entry decisions, mergers, or acquisition. Empirical support for the countervailing effects of some drivers is weak (Stahl and Voigt 2008), as studies tend to view effects in isolation, rather than simultaneously. Our research aims to consolidate and reconcile the interlinked effects of the drivers of international market entry decisions by considering their effects mediated through market potential. These questions are also important to managers for appropriate resource allocation decisions toward countries with better potential market outcomes. Thus, we may show managers that some drivers of market entry have countervailing effects and can both advance and retard entry timing. The mediation approach also enables us to better understand the dual effects of some drivers, overcoming some of the limitations of prior theory-building efforts.

We address this gap in the literature using a causal mediation approach where we decompose the total effects of the key drivers of market entry into direct and indirect (mediation) effects through market potential. To capitalize on this investigation, we must apply the insights gained to the determination of market entry timing. Thus, we show the importance of these drivers in the identification of target segments and demonstrate their predictive ability in allocating a country to a particular target segment. An effective approach to market selection is the use of demand- and supply-side variables to segment countries and then use segment-level measures to determine market entry timing (e.g., Sood, James, and Tellis 2009). The identification and targeting of market segments are critical to the success of most types of new product strategies for a multinational enterprise operating in the global marketplace (Katsikeas 2018). IM literature suggests the empirical validation of segments for targeting a new sample of countries (Papadopoulos and Martin 2011; Wedel and Kamakura 2002), testing the predictive ability of segment solutions (Wedel and Kamakura 2002), and using theory-driven approaches instead of exploratory analysis to aid management decision making (Steenkamp and Ter Hofstede 2002).

To summarize, we investigate two research questions:

1. How are the main drivers of international market entry mediated by market potential?
2. Given a set of countries optimally partitioned into segments for market entry timing, do the main drivers of international market entry hold predictive ability to validate these segments for targeting?

To investigate our research questions, we use a multinational data set containing 274 market entry timings by 28 major international market operators (e.g., Orange, Vodafone) into the mobile broadband markets of 79 countries. The market entry timing is defined as the time from the international launch of a technology (e.g., 4G) until a telecom operator uses that technology to enter a new country (Meade and Islam 2021). We address our first research question using a causal mediation approach and our second question using recent advances in machine learning procedures.

We offer theoretical, methodological, and managerial contributions to the field of IB/IM:

- *Theoretical.* We establish *how* key drivers of market entry decisions are related both conceptually and operationally. We decompose the total effects of the key drivers into direct and indirect (i.e., mediated) effects through market potential, using a causal approach to give deeper insights into the underlying process.
- *Methodological*. Using recent advances in machine learning, we develop a classification model using the key market entry drivers to validate previously identified latent segments. The model is useful to inform market entry decisions in cases where no prior data on market demand are available (e.g., new markets, markets soon after product launch) to predict segment membership.
- *Managerial.* Our findings of full or partial mediation of the key drivers by market potential indicate that managers would benefit from using market potential available from early demand data to decide on market entries. In the absence of such demand data, macroeconomic attractiveness is the most important determinant, by far, of the entry decision.

We present our work in the following sections: background literature and formulation of hypotheses, methodology, and results, followed by a discussion of the implications of this work and suggestions for further research.

## Background Literature and Hypothesis Development

### Background Literature

**Figure 1.**
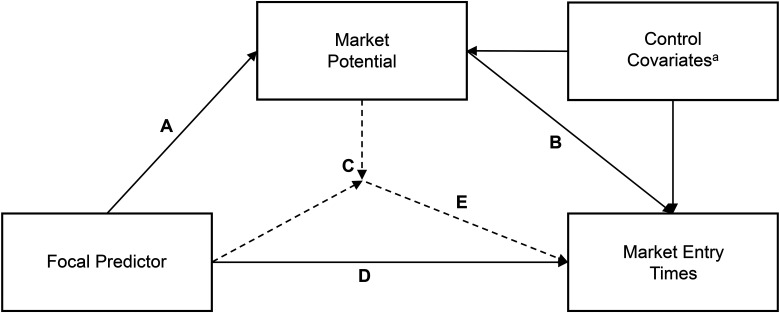
A conceptual framework decomposing the total effect of a driver of entry timing into direct and indirect effects.

^a^Main drivers except focal predictor.

*Notes*: Each of the four main drivers of market entry timing is taken as a focal predictor. The labels A, B, C, D, and E represent the relevant regression coefficients. The direct effect of the focal predictor is (D + EC) and the pure indirect effect via the mediator of market potential is (AB).

Both the IB and IM literature emphasize the importance of investigating the process through which the main drivers, such as macroeconomic attractiveness, impact the outcome variable (here, market entry timing) (Islam and Meade 2018; Zachary et al. 2014). Our objective is to decompose the impact of the key drivers of market entry timing into direct and indirect, or mediated, effects. A mediating variable (in our case, market potential) may be responsible for some or most of the effect of the driver on the outcome (in our case, market entry timing). The conceptual framework in Figure 1 summarizes our approach to the first research question. We consider each key driver as the focal predictor and decompose its total effects on market entry timing into a direct effect and an indirect (mediating) effect through market potential after controlling for other factors. Next, we discuss the mediating variable, market potential, and then discuss the main drivers of market entry.

Market potential is the size of the market for a specific technology/product measured as the total sales over the long run that can be obtained from market entry throughout the product’s presence in the market (Gaston-Breton and Martin 2011; Robertson and Wood 2001). Zachary et al. (2014) review the literature of the previous 25 years and suggest that a country's market potential is a major driver of market entry timing. It is widely recognized as a key predictor of market selection and a driver of company expansion in foreign markets (see Robertson and Wood 2001; Sakarya, Eckman, and Hyllegard 2007). Foreign direct investment theory proposes that firms enter international markets with the expectation that the benefits exceed the costs incurred (Vernon 1966), and the benefits exist largely due to the large market size or potential of the target country (Davidson 1980; Dunning 1998; Ellis 2008). Market potential is recognized as the most important host country characteristic in market entry decisions (Ellis 2008; Kobrin 1976; Terpstra and Yu 1988). Samli (1977) used multiple macroeconomic indicators as indirect measures of market potential until technology-specific measures of market potential became available. While other key drivers of market entry timing, such as macroeconomic attractiveness, social heterogeneity, market concentration, and population density, precede and drive market-specific potential, market potential is considered an exogenous rather than an endogenous or mediating variable in much of the literature.

The first main driver, macroeconomic attractiveness, is a construct that describes a country's wealth and prosperity. The literature identifies country innovativeness, globalization, economic prosperity, and standard of living as the key components of macroeconomic attractiveness (Ekeledo and Sivakumar 1998; Gaston-Breton and Martin 2011; Sakarya, Eckman, and Hyllegard 2007). Country innovativeness is evidenced by various factors including the level of innovation input and output, institutions, cumulative technological sophistication, human capital and research, infrastructure and knowledge, technology, and creativity in a country (Furman, Porter, and Stern 2002; Islam and Meade 2015; Tellis, Yin, and Bell 2009). Globalization captures how a country's individuals or firms interact with other parts of the world to exchange information (Oxley and Yeung 2001). Countries with higher social globalization tend to have a greater likelihood of accepting and adopting new products (Tellis, Stremersch, and Yin 2003). New technology diffuses rapidly when countries interact through economic globalization and international trade (Dreher 2006; Gatignon, Eliashberg, and Robertson 1989). Standards of living reflected in indicators of economic prosperity such as personal consumption and flow of information or funds are positively associated with the speed of diffusion of new products (Baliamoune-Lutz 2003; Dekimpe, Parker, and Sarvary 2000; Lee, Hong, and Hwang 2017; Perkins and Neumayer 2005; Van Everdingen, Fok, and Stremersch 2009). Research in entry timing has moved from “first mover” in an attractive market to a more pragmatic focus on timing the entry decision to gain an entry-timing advantage (e.g., Bohlmann, Golder, and Mitra 2002). These advances create a need for an integrative framework to shed light on how macroeconomic attractiveness impacts the timing of entry decisions.

The second main driver, market concentration, reflects the competitive structure of a market (e.g., Madden and Savage 2000) and is calculated by summing the squares of the market share of each firm competing in a market. The industrial organization literature has examined the role of market structure and the level of competition in the market entry decision (Katz and Shapiro 1992). When considering the decision to enter a profitable market with growing demand, a firm wants to enjoy an advantage such as low competitive intensity (Choudhury and Khanna 2014). Javalgi et al. (2011) identify, among others, the level of competition as the second-most-important driver, following market demand attractiveness. Early industrial organization literature suggested that there is an inverted U-shaped relationship between market competition and the optimal number of market entries (see Dixit and Stiglitz 1977; Salop 1977) that arises from the countervailing effects of competition.

The third main driver, social heterogeneity, is a measure of a target country's ethnic and cultural diversity and an important predictor of economic performance (see Easterly and Levine 1997; Luiz 2015). Ethnic diversity includes cultural features such as language, customs, religion, and a shared group history (Fearon 2003). Cultural dimensions (such as Hofstede's individualism–collectivism, power distance, uncertainty avoidance, and masculinity–femininity) have been found to influence market entry timing (e.g., Dekimpe, Parker, and Sarvary 1998; Gupta, Pansari, and Kumar 2018; Kirkman, Lowe, and Gibson 2017; Takada and Jain 1991; Talukdar, Sudhir, and Ainslie 2002; Van Everdingen, Fok, and Stremersch 2009). Stahl and Tung (2015) find a tendency to “over-emphasize the liabilities associated with cultural differences while de-emphasizing the potentially positive role of cultural diversity” (p. 393). They find negative effects of social heterogeneity reported in 53% of studies, and either positive or mixed or inconclusive results in the remaining studies. They recommend integrating the positive and negative effects of social heterogeneity in future research.

The fourth main driver, population density, reflects the concentration of potential demand and generates the linkages, the infrastructure, and the effective market size for innovation and adoptions (Van Everdingen, Fok, and Stremersch 2009). Population density is measured by dividing the population size of the country by its geographical size in square kilometers (Aarstad, Kvitastein, and Jakobsen 2016). Klasen and Nestmann (2006) find an inverted U-shaped relationship between population density and growth potential, indicating two latent countervailing forces of population density, one positive and one negative (Haans, Pieters, and He 2016). Countries with dense population are more susceptible to foreign influence, and information about innovation easily penetrates the social system (Mitra and Golder 2002; Van Everdingen, Fok, and Stremersch 2009). This is particularly relevant for countries with low levels of technology. The countervailing effects of slowing technology diffusion due to higher population density arises from increased cost of information to the consumers.

Two of the drivers, macroeconomic attractiveness and social heterogeneity, are formative constructs, each measured by several variables. We discuss details of the operationalization of these constructs and their component variables in the “Methodology” section.

### Hypothesis Development

Next, we develop hypotheses on the mediating effect of market potential on the relationship between the main drivers of market entry decisions and market entry timing.

#### Macroeconomic attractiveness

Countries with greater macroeconomic attractiveness possess advantages that make early decisions to enter more profitable than in less attractive countries (Bell and Pavitt 1993). Economic prosperity enables consumers to take higher risks to adopt new products, and people with higher incomes tend to purchase a new product earlier. At the country level, economic wealth enables a nation to take higher risks to invest in infrastructure to facilitate adoption of a new technology. Firms in economically attractive countries benefit from skilled labor, high capital–labor ratios, low interest rates, and sufficient financial resources to absorb any losses (Islam and Meade 2018). These advantages make early-entry decisions more profitable in economically attractive countries than in less attractive countries (Bell and Pavitt 1993).

The literature also shows that macroeconomic prosperity provides microeconomic stability, opening the economy to external markets and making products and services more affordable to consumers (Shane 1993; Talukdar, Sudhir, and Ainslie 2002), leading to increased market potential. Thus, we test two hypotheses concerning the direct and indirect effect of macroeconomic attractiveness.

**H_1a_**: Indirect effect: Macroeconomic attractiveness reduces market entry timing through market potential, such that market potential partially mediates the relationship between macroeconomic attractiveness and market entry timing.
**H_1b_**: Direct effect: Macroeconomic attractiveness reduces market entry timing such that a market with higher macroeconomic attractiveness has shorter market entry timing than markets with lower macroeconomic attractiveness.

#### Market concentration

The literature shows that higher market concentration influences the speed of adoption in the existing target market and expands market potential. On the one hand, market competition is a primary driver of price decline, making innovations economically attractive to a wider population (Feichtinger 1982; Kalish 1983; Mahajan and Peterson 1978). The presence of a few large firms enhances the effects of experience and economies of scale, leading to faster price decline (Bayus, Kang, and Agarwal 2007; Kim, Bridges, and Srivastava 1999; Lambkin and Day 1989). Large firms often have lower financial costs, enabling them to acquire specialized machinery and manpower to increase efficiency. Thus, higher market concentration (equivalent to low competitiveness) can both increase the speed of adoption in the existing target market and expand the market potential as new products become affordable to more people.

On the other hand, market domination by a few firms exploiting their economies of scale creates entry barriers for new firms and impedes innovation (Li and Liu 2013; Porter 1980). Control of critical resources by a few incumbents also increases the likelihood of failure for new firms (Carroll 1985). Changes in market concentration signal the extent to which new market operators increase or decrease their control of critical resources and thus reflect new entrants’ chances of entering and thriving (Li and Liu 2013).

Given these two countervailing effects of the focal predictor market concentration, we hypothesize that the total effects of market concentration decompose into direct and indirect effects as follows:

**H_2a_**: Indirect effect: Market concentration reduces market entry timing through market potential, such that market potential partially mediates the relationship between market concentration and market entry timing.
**H_2b_**: Direct effect: Market concentration increases market entry timing such that a more concentrated market increases market entry timing compared with a less concentrated market.

#### Social heterogeneity

As with market concentration, social heterogeneity also deters and enhances market entry timings. The deterrence occurs for the following reasons. New technologies diffuse more slowly in countries with a more mixed population (Takada and Jain 1991), and this slow growth and low market potential deter market selection (Folta and O’Brien 2004; Sakarya, Eckman, and Hyllegard 2007; Zachary et al. 2014). Social heterogeneity increases the transaction costs of doing business across nations (Berry, Guillén, and Zhou 2010), negatively impacts the probability of market entry (Folta and O’Brien 2004; Markman et al. 2019), and hinders learning across groups in the overall population (Alesina et al. 2003; Islam and Meade 2018; Rogers 1995). Social heterogeneity is also associated with interventionism, lower government efficiency, more corruption, and lower infrastructure quality. Fragmented societies are more prone to poor policy management and pose more politicoeconomic challenges, thereby deterring foreign entry to the country (La Porta et al. 1999) as all these factors inhibit growth and market potential.

In contrast, Alesina and La Ferrara (2005) argue that a diverse ethnic mix brings a variety of ability, experience, and culture that facilitates innovation and creativity, which leads to faster market growth. A growing body of literature finds cultural diversity beneficial to global corporations (Insch and Miller 2005; Kostova and Zaheer 1999; Mezias 2002)—for example, in foreign market entry decisions, joint ventures, and mergers and acquisitions (Brannen 2004; Sarala and Vaara 2010). Østergaard, Timmermans, and Kristinsson (2011) find that social heterogeneity drives innovation and fosters new product diffusion; they suggest that expatriates in leading firms and market operators play an important role in technology transfer, giving access to knowledge and accelerating innovation adoption. These conditions positively impact earlier entry to growth markets (Kerr 2008).

Due to these countervailing effects, we hypothesize that the total effects of social heterogeneity decompose into direct and indirect effects as follows:

**H_3a_**: Indirect effect: Social heterogeneity increases market entry timing by reducing market potential, such that market potential partially mediates the relationship between social heterogeneity and market entry timing.
**H_3b_**: Direct effect: Social heterogeneity reduces market entry timing, such that markets with higher heterogeneity exhibit reduced market entry timing compared with markets with lower social heterogeneity.

#### Population density

Densely populated countries are attractive to international market operators as they offer economies of scale, low transportation costs, easy access to large market potentials, stability in demand, and easy access to human capital (Krugman 1991). The takeoff probability of a new product is higher in densely populated areas, as high density enhances the speed at which an innovation diffuses through the wider population (Dekimpe, Parker, and Sarvary 2000). Islam and Meade (2015) incorporated population density in market potential as a coefficient of the Bass model while modeling firm-level multinational market penetrations of mobile phones and found that market potential increases with higher population density.

However, if population density becomes too high, the consumer's cost of selecting the right information increases, thus lowering the benefit of faster knowledge transfer and thereby slowing the speed of diffusion (Klasen and Nestmann 2006). Islam and Meade (2018) find a nonsignificant negative effect of population density on introduction times and sales takeoff times for four generations of mobile phones. This effect is partly because densely populated areas occur in low- or lower-middle-income countries where many people are unable to afford a price that is profitable to the market operator.

Because there are two countervailing influences, we decompose of total effects of population density into direct and indirect effects as follows:

**H_4a_**: Indirect effect: Population density decreases market entry timing by increasing market potential, such that market potential partially mediates the relationship between population density and market entry timing.
**H_4b_**: Direct effect: Population density increases market entry timing, such that markets with higher density exhibit increased market entry timing compared with markets with lower population density.

## Methodology

Here, we describe the methodological steps used to investigate our research questions. First, we describe variables, data sources, and operationalization of the main drivers. Second, we describe the growth-mixture-modeling (GMM) approach used to estimate each country's market potential and to segment the countries into homogeneous clusters. Third, we explain a causal-mediation approach to decompose the total effects of the main drivers of market entry timing into direct effects and indirect effects via market potential. Fourth, we describe our approach to using the main drivers of market timing to validate the segmentation, thus providing a tool for managers to segment new countries not used in the calibration process.

### Variables and Data Sources

We describe our dependent variable, market entry timing, followed by the key constructs and control variates in the model summarized in Figure 1.

#### Market entry timing: dependent variable

We use 274 market entry timings by 28 major international market operators (e.g., Orange, Vodafone) for 79 countries. We obtained this data set from GSMA Intelligence. The market entry timing in years is the time from international launch of a technology (December 2009 for 4G) until a telecom operator uses that technology to enter a new country (Meade and Islam 2021).

#### Main drivers

The four main drivers of market entry timing are identified in the “Background Literature” subsection. Each driver is used as a focal predictor in the mediation analysis for research question 1 and for the validation of the segmentation for research question 2. We use data from 2009, the prelaunch year of the international launch of 4G.

#### Macroeconomic attractiveness

This driver is a formative construct formed from four subconstructs: country innovativeness, globalization, standard of living and civil liberties, and economic prosperity. Each subconstruct is derived from observable variables.

We measure country innovativeness using the overall index and the innovation input and output subindices to assess aspects of a country's environment conducive to innovation. The innovation input subindex measures institutional environment, human capital and research, information and communications technology infrastructure, and market and business sophistication. The innovation output subindex measures the output of knowledge and technology (e.g., patent applications, scientific and technical published articles in peer-reviewed journals), and creative outputs (e.g., trademark registration). We use three measures of globalization, the overall index and the economic and social subindices. The overall index combines the economic, social, and political dimensions of the globalization. It encompasses trade, foreign direct investment, portfolio investment, and income payments to foreign nationals as percentages of gross domestic product (GDP). The economic globalization subindex includes the flows of goods, capital, and services. The social globalization subindex includes the spread of ideas, images, information, and people. We measure the economic prosperity of a country using annual data on the purchasing-power-adjusted GDP per capita. We use the Human Development Index to measure the standard of living. It reflects life expectancy, years of schooling, gross national income per capita, purchasing power parity, and civil liberties (based on data on equality of opportunity, freedoms of expression and religion, and level of education). In Table 1, we list the subconstructs with their observable variables, their data source and supporting references.

**Table 1.** Supporting Information for the Macroeconomic Attractiveness and Social Heterogeneity Constructs.

**Dimensions**	**Reference**	**Data Source^a^**	**Observed Covariates**	**Loading**
**Macroeconomic Attractiveness**				
Country innovativeness	Furman, Porter, and Stern (2002), Islam and Meade (2015), Tellis, Yin, and Bell (2009)	Global Innovation Index (Cornell University, INSEAD, and WIPO 2014)	Innovation index	.363
			Innovation input subindex	.351
			Innovation output subindex	.348
Globalization	Dreher (2006), Gatignon, Eliashberg, and Robertson (1989), Islam and Meade (2015), Talukdar, Sudhir, and Ainslie (2002), Tellis, Stremersch, and Yin (2003)	KOF Globalization Index	Globalization	.359
			Economic globalization	.329
			Social globalization	.357
Standard of living and civil liberties	Baliamoune-Lutz (2003), Dekimpe, Parker, and Sarvary (2000), Lee, Hong, and Hwang (2017)	United Nations Development Program; freedomhouse.org	Human Development Index	.331
			Civil liberties	−.258
Economic prosperity	Dekimpe, Parker, and Sarvary (2000); Perkins and Neumayer (2005); Van Everdingen, Fok, and Stremersch (2009)	Penn World Table	National income GDP/cap.	.288
**Social Heterogeneity**				
Cultural heterogeneity	Dekimpe, Parker, and Sarvary (1998), Fearon (2003), Perkins and Neumayer (2005),Takada and Jain (1991), Talukdar, Sudhir, and Ainslie (2002)	Fearon (2003)	Cultural fractionalization	.707
Ethnic heterogeneity			Ethnic fractionalization	.707

^a^ Data for observed covariates of macroeconomic attractiveness from 2009.

#### Social heterogeneity

This driver is also a formative construct formed from measures of cultural and ethnic heterogeneity or fractionalization. Ethnic fractionalization is the probability that two individuals selected at random from a country will be from different ethnic groups. Cultural fractionalization is measured similarly using cultural features such as language, customs, religion, and a shared group history. Table 1 provides details.

Both macroeconomic attractiveness and social heterogeneity are formative constructs (see Edwards 2011), and the observation of these constructs for each country is estimated by the first component of a principal component analysis (the loadings are given in the right-hand column of Table 1). The proportion of variation explained by the first principal component is 78% for macroeconomic attractiveness and 88% for social heterogeneity.

#### Population density

This driver is identified by Klasen and Nestmann (2006) and Van Everdingen, Fok, and Stremersch (2009). The data source is Penn World Table 2009.

#### Market concentration

This driver is measured using annual data on Herfindahl–Hirschman Index (HHI) for each country, and the data source is GSMA Intelligence 2009. HHI is a commonly accepted measure of market concentration and is calculated by squaring the market share of each firm competing in a market and then summing the resulting numbers. Thus, it takes into account the relative size of the firms in a market. The closer a market is to a monopoly, the higher the market's concentration (and the lower its competition), and the higher the HHI. It is lower when the market is occupied by a large number of firms of relatively equal size.

### Control Covariates

Multidimensional distance between the domicile of market operators and the host country has been used as a control in an analysis of the determinants of the market entry decision (Sood and Kumar 2018). The underlying assumption is that distance introduces friction and complexity (Vermeulen and Barkema 2002), increases transaction costs, prevents the flow of information (Johanson and Vahlne 1977), and deters market entry. We include geographic distance, knowledge distance, and global connectedness distance as suggested by Berry, Guillén, and Zhou (2010). The data source is the Lauder Institute, University of Pennsylvania. We also include anticipated growth rate as a control variable. This variable was identified in prior literature to influence market entry timing, especially at the screening stage (Folta and O’Brien 2004; Sakarya, Eckman, and Hyllegard 2007; Zachary et al. 2014). In line with previous researchers (Bass 1969; Kovach 2018), we operationalize growth rate as the time to reach peak demand, available from our GMM analysis.

### GMM: Mediator and Latent Segments

GMM uses structural equations both to capture the behavior of individuals over time and to segment individuals into relatively homogeneous groups (Bassi 2016; Bollen and Curran 2006; Grimm and Ram 2009; Ram and Grimm 2007; Singer and Willet 2003). For GMM, we use annual market-penetration data from GSMA Intelligence for mobile 4G broadband (launch date December 2009) for 130 countries from 2010 to 2016. Our use of GMM fulfills two roles simultaneously, enabling us to estimate the parameters of the Bass model to describe the adoption of 4G technology by consumers in different countries of diffusion and to allocate countries to latent segments based on the similarity of the parameters. Bass (1969) models the diffusion of an innovation using three parameters, the coefficients of external and internal influence and market potential; thus, GMM provides observations of the mediating variable, market potential, for our response to research question 1. The latent segments are formed based on the similarity of the trajectory of a country's adoption of 4G technology. As part of answering research question 2, we compare market entry times within the different segments.

Using the annual market-penetration data, we see that the diagnostics of GMM indicate that the variation among the diffusion rates of mobile 4G broadband in 130 countries is best represented by four latent segments, following the model selection procedure by Nylund, Asparouhov, and Muthén (2007). This analysis also provides the estimates of market potential used in the mediation analysis. Table 2 summarizes the four segments.

**Table 2.** Summary of Income Level, Region, and Bass Model Parameters for the Four Segments Identified by GMM.

**Segment**	**A**	**B**	**C**	**D**
Percentage of 130 countries	10.0	28.5	25.4	36.2
Number of countries	13	37	33	47
High income	12	26	11	
Upper middle income		9	14	9
Lower middle income		2	8	22
Low income	1			16
Africa	1	2	7	20
Americas	1	2	8	11
Asia	7	6	9	14
Europe	3	26	8	1
Oceania	1	1	1	1
Market potential: mean	8.93	7.72	5.55	3.13
Market potential: SD	3.12	2.09	2.59	2.00
Coeff. external influence	.116	.063	.029	.009
Coeff. internal influence	.370	.266	.401	.594
Time to reach peak	2.39	4.38	6.11	6.95

**Figure 2.**
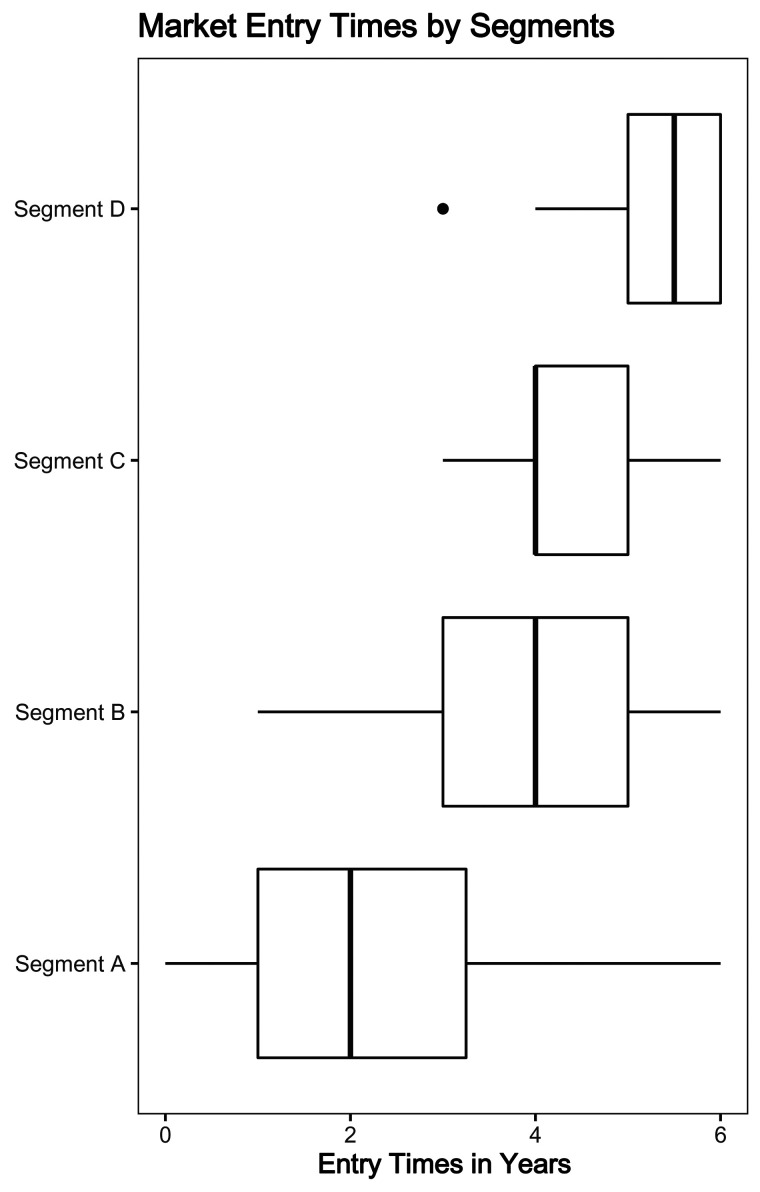
A box plot of market entry times for 79 countries, within each segment.

The segments are labeled according to their market potential in decreasing size; in addition, the time to reach peak adoption (a function of only the coefficients of external and internal influence) appears in increasing number of years. Thus, segment A has the highest market potential, 8.93 out of 10 consumers, and the shortest time to reach peak adoption, 2.4 years. The estimated coefficients of external influences (Mahajan, Muller, and Srivastava 1990) indicate the propensity of the countries in each segment to respond to marketing effort, thereby potentially aiding international market operators in their resource planning. As we expected, economic sectors are a partial explanation of the classification into segments, with the regional composition of the segments closely related to the distribution of income levels. These findings highlight the significant advantages of GMM in segmenting the countries in an international market under the limitation of insufficient or very early sales data. However, these segments are predicated on predicted adoption, with market potential as a prominent component. In Figure 2, we show a box plot of the 274 observed entry times for the subset of 79 countries where data are available. There is a strong link between segment membership and market entry times, looking at the left-hand side of each box, which denotes the lower quartile of entry times; we see that this lower quartile increases by at least six months as we move from segment to segment.

### Methods of Analysis

#### Measuring the mediation effect of market potential

A mediator describes the mechanism by which a key driver (e.g., economic attractiveness) affects the final stage of the outcome variable (market entry timing). A mediating variable (market potential) may be responsible for some of the effect of the cause on the outcome, to paraphrase VanderWeele (2014). We use a causal-mediation approach to measure association with our nonexperimental observational data (see, e.g., Quandt 1972). This approach allows us to decompose the total effects of the drivers of market entry timing into direct and indirect effects, giving a deeper insight into the underlying process (Morgan and Winship 2015; Preacher 2015; VanderWeele 2014). We summarize our conceptual approach in Figure 1. The mediation effect of market potential on each of the four main drivers of market timing (macroeconomic attractiveness, market concentration, social heterogeneity, and population density) is tested individually. The driver being tested is called the focal predictor. We follow the approach of Baron and Kenny (1986) with two further assumptions: (1) no interaction between macroeconomic attractiveness and market potential on the market entry times and (2) no unmeasured confounders, in order to claim causality. This approach involves running two regressions (Muthén and Asparouhov 2015):

1. Market entry timing as a function of the focal predictor, market potential, their interaction (focal predictor × market potential), and the control covariates.
2. Market potential as a function of the focal predictor and the control covariates.

#### Validating the segmentation using a classification approach

The countries are segmented by GMM according to their diffusion trajectories, including market potential. Here, we develop a classification model using the main drivers of market entry timing to determine segment membership. Once the classification model has been calibrated using a sample with known segment membership, the model can be used to target a segment for an unseen sample of countries for market entry decisions.

We use random forests, a machine-learning algorithm, as our classification algorithm. Machine learning methods are “an exciting and underutilized toolkit for strategy and management researchers” (Choudhury, Allen, and Endres 2021, p. 31). Random forests is an ensemble technique based on the use of a set of small classification and regression trees (Breiman et al. 1984). It approximates functions smoothly by averaging over the step-functions of single trees. This property allows random forests to better capture nonlinearities and complex interactions (Strobl, Malley, and Tutz 2009). To classify a country, the variables are fed into each tree in the forest, resulting in each tree “voting” for a segment; the segment with the most votes is the choice of the random forest. For further details about random forests, see Lin and Jeon (2006); Biau, Devroye, and Lugosi (2008), and Zhao and Hastie (2021), who all discuss the role of machine learning in causal inference.

### Results

#### The mediation effect of market potential

We estimate the direct and indirect effects of the focal predictors on market entry timing, mediated by market potential, as shown in Figure 1. Each of the four main drivers of market entry timing is used as the focal predictor, and the effects are estimated by regression. Table 3 shows the estimates of the indirect effect (AB) and the total direct effect (D + EC) for each of the main drivers.

**Table 3.** Estimates of Indirect and Direct Effects of the Four Main Drivers of Market Entry Timing.

**Focal Predictor**	**Decomposition of Total Effect**	**Estimate**	***p*-Value**
Macroeconomic attractiveness	H_1a_: Pure indirect effect, AB < 0	−.350	<.001
	H_1b_: Total direct effect, D + EC < 0	−.191	.146
Market concentration	H_2a_: Pure indirect effect, AB < 0	−.186	.008
	H_2b_: Total direct effect, D + EC > 0	.560	.012
Social heterogeneity	H_3a_: Pure indirect effect, AB > 0	.099	.010
	H_3b_: Total direct effect, D + EC < 0	−.309	.012
Population density	H_4a_: Pure indirect effect, AB < 0	−.020	.087
	H_4b_: Total direct effect, D + EC > 0	.168	.016

In support of our mediation hypothesis H_1a_, we find that the pure indirect effect of macroeconomic attractiveness on entry timings is negative (greater attractiveness reduces entry times) and significant (at 5% unless stated otherwise). However, H_1b_ is not supported, as the direct effect of macroeconomic attractiveness is not significant. Thus, we find full mediation—a significant indirect effect of macroeconomic attractiveness—when a market potential estimate is available.

We hypothesize that market concentration has two counteracting impacts on market entry decisions. As an indirect effect (H_2a_), it reduces entry times by driving a steeper price decline, increasing market potential. As a direct effect (H_2b_), it lengthens entry times by creating an entry barrier. Our mediation hypothesis H_2a_ is supported: the indirect effect of market concentration on entry timing is significantly negative, with entry timings shortened as concentration is increased. H_2b_ is also supported: there is a significant positive direct effect as market concentration lengthens market entry timings. The indirect effect acting in the opposite direction to the direct effect is called competitive mediation by Zhao, Lynch, and Chen (2010).

Does social heterogeneity have two counteracting impacts on market entry decisions? Our hypothesis of a positive indirect effect (H_3a_) is supported: social heterogeneity lowers market potential due to slow growth lengthening entry times. Our hypothesis of a negative direct effect (H_3b_) is also supported: social heterogeneity reduces entry times by accelerating innovation adoption, driven mainly by expatriates filling roles in leading firms and market operators.

Does population density also have two counteracting effects on market entry timing? H_4a_ is marginally supported, as we find a significant (at 10%) negative indirect effect: a higher population density reduces market entry timing by increasing market potential. H_4b_ is also supported, as we find a significantly positive direct effect: countries with higher population density have longer entry times, possibly because high density is associated with lower incomes.

Sensitivity analysis following Ding and VanderWeele (2016) showed that this finding is not sensitive to violations of the assumption of no unmeasured confounding variables. For all four drivers, we find that market potential has a significant mediating effect on market entry timing. The drivers macroeconomic attractiveness, market concentration, and population density tend to increase market potential, which shortens market entry times. In contrast, social heterogeneity tends to lower market potential and can lengthen market entry times.

#### Validating the latent segments

In the absence of adoption data for a country, the classification approach will facilitate market entry decision making if the segment membership can be predicted. To achieve this, we validate the segment profiles by testing how well the main drivers of market entry timing—macroeconomic attractiveness, social heterogeneity, market concentration, and population density—predict segment membership for each country. Further, to answer our research question 2, we evaluate the relative importance of these drivers and their predictive ability in determining segment membership.

We divide the sample into 70% training data and 30% testing data (Choudhury, Allen, and Endres 2021). The model is fitted to the training data, and both in-sample and out-of-sample predictions are made. The results from a sample run of random forests are shown in Table 4. Within the training set, all the countries were correctly allocated to their segment, and within the test set three-quarters of the countries were correctly allocated. In all but one case where a country is misallocated, the country is put into an adjacent segment. Over all runs, the predictive performance is 96.5% correct allocations in-sample and 72.3% out-of-sample. Thus, the machine learning approach of random forests has a high degree of accuracy in using the four drivers to allocate a country to its appropriate segment and consequently determine timing of market entry effectively.

**Table 4.** A Summary of the Accuracy of a Sample of a Random-Forest Prediction of Segment Membership.

		**Segment Membership Generated by GMM**				
		**Segment A**	**Segment B**	**Segment C**	**Segment D**	**Total**
Predicted membership generated by random forest	Segment A	10	0	0	0	10
		3	0	0	0	3
	Segment B	0	26	0	0	26
		0	10	0	1	11
	Segment C	0	0	24	0	24
		0	1	7	1	9
	Segment D	0	0	0	33	33
		0	1	2	11	14
	Total	10	26	24	33	93
		3	12	9	13	37

*Notes*: For each cell, the upper entry is for the training set and the lower entry is for the test set. For this run, classification accuracy is 100% for the training set and 75.67% for the test set.

#### Relative importance of the four drivers in validating segment membership

**Figure 3.**
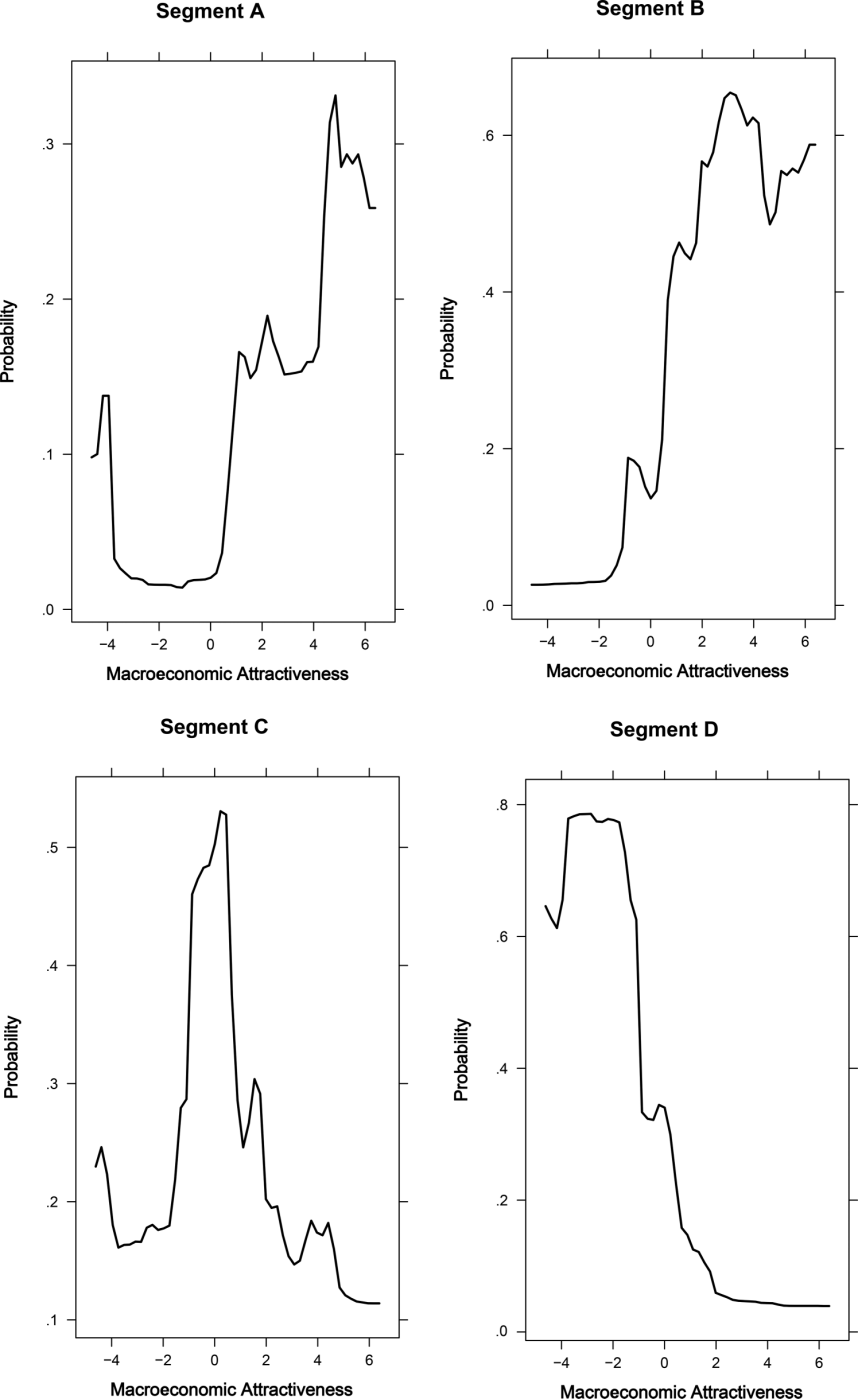
Partial dependence plots of macroeconomic attractiveness by segments.

To determine the relative predictive accuracy of the four drivers, we use the mean decrease in impurity, a measure of relative importance suggested in recent literature (Choudhury, Allen, and Endres 2021; Zhao and Hastie 2021). Mean decrease in impurity assesses the decrease in accuracy when a driver is omitted, indicating how important that driver is in segmenting the data correctly. For our random-forests analysis, we find the following relative importance: macroeconomic attractiveness, 42.7%; market concentration, 14.4%; social heterogeneity, 17.1%; and population density, 18.0%. Thus, macroeconomic attractiveness is more than twice as important as any of the other drivers in the model. A partial dependence plot is a visualization tool proposed by Friedman (2001), showing the relationship between the driver and the probability of a country being allocated to a segment. In Figure 3, we show example plots for macroeconomic attractiveness. We see that a positive score for market attractiveness is associated with a high probability of assignment to segments A and B, with shorter market entry times, and that a negative score is associated with a high probability of assignment to segment D, with longer market entry times.

## Discussion

For the first research question, our analysis of international market entry timings in mobile broadband markets confirms the mediating impact of market potential between the main drivers of the market entry decisions and market entry timings. Having relaxed the assumption that all drivers are exogenous (as typically assumed in the literature), we used a causal-mediation approach to gain insight into the underlying process and to capture counteracting effects. We developed hypotheses that decomposed the effect of the four main drivers into direct and indirect effects. We found support for an indirect effect of macroeconomic attractiveness on market entry timings via market potential (H_1a_), but not a direct effect of macroeconomic attractiveness (H_1b_). This full mediation highlights the indirect role of macroeconomic attractiveness at a product postlaunch stage when the data-based market potential is available. The counteracting effects of market concentration (H_2a_ and H_2b_), social heterogeneity (H_3a_ and H_3b_), and population density (H_4a_ and H_4b_) are supported. Thus, seven out of eight of our hypotheses are supported.

We applied GMM to the diffusion data for mobile broadband for two reasons: (1) to obtain estimates of market potential for the countries in order to answer our first research question and (2) to allocate countries to segments with similar diffusion characteristics in response to our second question. We found that four segments, A to D, were appropriate. The 13 countries in segment A have an average market potential of 89% of the population and a lower quartile of market entry of one year. In contrast, the 47 countries in segment D have an average market potential of 31% of the population and a lower quartile of market entry of seven years.

To answer our second research question—what is the relative importance of the main drivers of market entry in predicting segmentation A to D?—we used random forests with the four main drivers of market timing as the classifying variables. Random forests achieved on average 96.5% accuracy for the training set (70% of the countries) and 72.3% accuracy with the test set (30% of the countries). We found that macroeconomic attractiveness was twice as important as any of the other drivers in determining segment membership. Next, we summarize our contribution and the implications of our study.

### Contribution to Theory and Methodology

#### Theoretical

We followed the building blocks of Whetten (1989) for contributing to a theory by considering (1) *how* variables in the model are related conceptually and operationally and (2) *what* the underlying mechanisms are that connect the causal link. Contrary to implicit assumptions in the literature that all drivers are exogenous, we show that market potential is endogenous. It is a mediating variable between the main drivers of market entry and market entry timing and thus offers deeper insights into the underlying process. We decomposed the counteracting impacts of market concentration, social heterogeneity, and population density on market entry timing to uncover opposing direct and indirect effects. Thus, we established a competitive mediation effect (Zhao, Lynch, and Chen 2010) for these three drivers.

#### Methodological

We develop a classification model to validate latent segments using the main drivers of market entry as predictors with a machine learning procedure. Our validated model can be used to predict segment membership for an unseen sample of countries to inform market entry decisions at product prelaunch or in the absence of early demand data for new products or new markets.

### Implications for Management

As input to the market entry decision, our finding of the full mediation of macroeconomic attractiveness by market potential suggests that managers should use demand-based estimates whenever possible and available. By pooling early multinational demand data via GMM, we provide international market operators with estimates of country-level market potential, a key determinant of market entry. We also provide segment-level growth potential and times to reach peak adoption. Our finding, in response to research question 2, indicates that in the absence of demand data, macroeconomic attractiveness is the most important determinant of the market entry decision, by far.

### Implications for Further Research

There are several implications of our findings for future research. We demonstrated countervailing effects of three out of four main drivers of market entry decisions. We established that two opposing effects additively generate the net effects of the drivers. Further research could explore multiplicative combinations of two such forces on entry decisions (Haans, Pieters, and He 2016). In this study, we do not consider spatial differences in our modeling framework, and each country is treated as a monolithic entity. Studies have shown some empirical support for spatial association in international segmentation (Hofstede, Wedel, and Steenkamp 2002). We found that the main drivers currently suggested by the literature led to only 72.3% accuracy of the classification model for out-of-sample predictions for the same product or a new product. Whether the misallocation of countries to segments can be attributed to random variation or a currently unobserved covariate is a matter for further research, which could also explore machine learning procedures, as they constitute an “underutilized tool kit for strategy and management research” (Choudhury, Allen, and Endres 2021, p. 30). Recent advances of machine learning procedures, such as a causal random forest, can be used to establish a causal relationship between the main drivers and the entry decision (Wager and Athey 2018). We showed how variables are conceptually and operationally related and demonstrated an underlying mechanism with the mediation; further research avenues include testing boundary conditions or contextual limitations with suitable moderators.
